# Multimarker synaptic protein cerebrospinal fluid panels reflect TDP-43 pathology and cognitive performance in a pathological cohort of frontotemporal lobar degeneration

**DOI:** 10.1186/s13024-022-00534-y

**Published:** 2022-04-08

**Authors:** Alba Cervantes González, David J. Irwin, Daniel Alcolea, Corey T. McMillan, Alice Chen-Plotkin, David Wolk, Sònia Sirisi, Oriol Dols-Icardo, Marta Querol-Vilaseca, Ignacio Illán-Gala, Miguel Angel Santos-Santos, Juan Fortea, Edward B. Lee, John Q. Trojanowski, Murray Grossman, Alberto Lleó, Olivia Belbin

**Affiliations:** 1 Hospital de La Santa Creu I Sant Pau, Universitat Autonoma de Barcelona, Barcelona, Spain; 2 Centre of Biomedical Investigation Network for Neurodegenerative Diseases (CIBERNED), Madrid, Spain; 3 Memory Unit and Biomedical Research Institute, IIB Sant Pau, c/Sant Quintí 77, 08041 Barcelona, Spain; 4 Penn FTD Center, Department of Neurology, Perelman School of Medicine at the University of Pennsylvania, Philadelphia, PA USA; 5 Penn Alzheimer’s Disease Research Center, Department of Neurology, Perelman School of Medicine at the University of Pennsylvania, Philadelphia, PA USA; 6 Center for Neurodegenerative Disease Research, Department of Pathology and Laboratory Medicine, Perelman School of Medicine at the University of Pennsylvania, Philadelphia, PA USA

**Keywords:** Calsyntenin-1, Frontotemporal lobar degeneration, Frontotemporal dementia, Cerebrospinal fluid, Biomarker, TDP-43, Tau

## Abstract

**Background:**

Synapse degeneration is an early event in pathological frontotemporal lobar degeneration (FTLD). Consequently, a surrogate marker of synapse loss could be used to monitor early pathologic changes in patients with underlying FTLD. The aim of this study was to evaluate the relationship of antemortem cerebrospinal fluid (CSF) levels of 8 synaptic proteins with postmortem global tau and TDP-43 burden and cognitive performance and to assess their diagnostic capacity in a neuropathological FTLD cohort.

**Methods:**

We included patients with a neuropathological confirmation of FTLD-Tau (*n* = 24, mean age-at-CSF 67 years ± 11), FTLD-TDP (*n* = 25, 66 years ± 9) or AD (*n* = 25, 73 years ± 6) as well as cognitively normal controls (*n* = 35, 69 years ± 7) from the Penn FTD Center and ADRC. We used a semi-quantitative measure of tau and TDP-43 inclusions to quantify pathological burden across 16 brain regions. Statistical methods included Spearman rank correlations, one-way analysis of covariance, ordinal regression, step-wise multiple linear regression and receiver-operating characteristic curves.

**Result:**

CSF calsyntenin-1 and neurexin-2a were correlated in all patient groups (*r*_*s*_ = .55 to .88). In FTLD-TDP, we observed low antemortem CSF levels of calsyntenin-1 and neurexin-2a compared to AD (.72-fold, *p* = .001, .77-fold, *p* = .04, respectively) and controls (.80-fold, *p* = .02, .78-fold, *p* = .02, respectively), which were inversely associated with post-mortem global TDP-43 burden (regression *r*^*2*^ = *.56, p* = .007 and *r*^*2*^ = *.57, p* = .006, respectively). A multimarker panel including calsyntenin-1 was associated with TDP-43 burden (*r*^*2*^ = .69, *p* = .003) and MMSE score (*r*^*2*^ = .19, *p* = .03) in FTLD. A second multimarker synaptic panel, also including calsyntenin-1, was associated with MMSE score in FTLD-tau (*r*^*2*^ = .49, *p* = .04) and improved diagnostic performance to discriminate FTLD-Tau and FTLD-TDP neuropathologic subtypes (AUC = .83).

**Conclusion:**

These synaptic panels have potential in the differential diagnosis of FTLD neuropathologic subtypes and as surrogate markers of cognitive performance in future clinical trials targeting TDP-43 or tau.

**Supplementary Information:**

The online version contains supplementary material available at 10.1186/s13024-022-00534-y.

## Background

A prominent feature of frontotemporal lobar degeneration (FTLD) is a region-specific degeneration and loss of synapses [1–4], which correlates with cognitive impairment [1]. Characterization of the changes in quantity and function of synaptic proteins and the relationship to pathologic abnormalities and clinical symptoms is essential to improve our understanding of early FTLD pathogenesis. An objective marker of synapse degeneration that is directly related to FTLD pathology would be invaluable for monitoring the progression of these early pathological changes, which could aid patient management, clinical trial recruitment and has potential as a measure of drug response in future clinical trials. Moreover, the clinical diagnosis of FTLD syndromes is challenged by the variable correspondence between the clinical syndrome and underlying neuropathological changes and there is a need for a biomarker that can distinguish neuropathologic subtypes. FTLD can be classified postmortem into four major neuropathological subtypes; FTLD-Tau (hyperphosphorylated tau inclusions), FTLD-TDP (transactive response DNA-binding protein-43; TDP-43), FTLD-FET (fused in sarcoma, Ewing’s sarcoma, TATA-binding protein associated factor 15) and FTLD-UPS (ubiquitin and p62) [2–4]. Here, we have focused on FTLD-Tau and FTLD-TDP, which represent 90–95% of FTLD cases.

The aim of this study is to a) compare the cerebrospinal fluid (CSF) profile of a panel of synaptic proteins in a neuropathological cohort of confirmed FTLD-Tau and FTLD-TDP, b) evaluate the relationship of antemortem CSF levels with postmortem Tau and TDP-43 burden and cognitive performance and c) determine their diagnostic accuracy. As reference groups we include cognitively normal controls where synapse degeneration is absent and Alzheimer’s disease (AD) where synapse degeneration is widespread. The proteins included in the study (calsyntenin-1, glua2, glua4, Neurexin-2a, Neurexin-3a, neuroligin-2, syntaxin-1b, thy-1 and VAMP-2) were brought forward from our previous study in clinical AD cohorts [5]. These proteins are enriched at the synapse [9] and are involved in a range of synaptic functions including pre-synaptic differentiation [6], dendritic spine assembly [11–14], postsynaptic Ca2 + signalling [7], AMPA receptor trafficking [16, 17], synaptic vesicle exocytosis [18–21] and synaptic plasticity [22–25].

## Methods

### Study design

This is a single-center cross-sectional study. Antemortem CSF samples obtained between 1992 and 2015 were retrospectively selected from samples at the Penn FTD Center and Alzheimer’s Disease Research Center (ADRC) at the University of Pennsylvania (Philadelphia, USA) [8, 9]. The study includes patients with FTLD-related syndromes followed to autopsy with a neuropathological diagnosis of FTLD-Tau (*n* = 24) or FTLD-TDP (*n* = 25) and patients with a clinical and neuropathological diagnosis of AD (*n* = 25). Patients included for study had prospective CSF collection as part of ongoing observational research programs at The Penn FTD Center aimed at developing biological markers that reflect pathophysiological changes in FTLD proteinopathies to improve antemortem diagnosis and prognostication. Patients underwent a Mini-Mental State Examination (MMSE) by neurologists masked to synaptic biomarker data within 5 months of the lumbar puncture. We also analyzed CSF samples from cognitively normal controls (*n* = 35) that were recruited from the community and screened by revising their medical and medication history. Controls self-reported a negative neurological and psychiatric history and were within the normal range of cognitive performance (Mini-Mental State Examination > 27). CSF samples from patients and controls were processed in the same way. Pre-analytical processing details for these CSF samples can be found elsewhere [8].

### Neuropathological classification and quantification of neuropathological burden

Neuropathological diagnosis was established following previously described methods and international published criteria [9–12]. Patients with a primary neuropathological diagnosis of Pick’s disease (*n* = 3), corticobasal degeneration (*n* = 7), progressive supranuclear palsy (*n* = 9), argyrophilic grain disease (*n* = 4) or non-classifiable non-AD tauopathies (*n* = 1) were classified as FTLD-Tau (4-repeat tauopathy; *n* = 20, 3-repeat tauopathy; *n* = 3, unclassified tauopathy; *n* = 1). Patients with TDP-43 inclusions were classified as FTLD-TDP (subtype A; *n* = 1, subtype B; *n* = 11, subtype C; *n* = 8, non-specified; *n* = 5). The majority of patients with FTLD-Tau (*n* = 20/24) and FTLD-TDP (n = 18/25) had a neurofibrillary tangle score of B0 or B1 in the National Institute on Aging-Alzheimer’s Association (NIA-AA) classification [26] and therefore had no evidence of significant AD comorbidity. All patients with a neuropathological diagnosis of AD had scores of B2 or B3 in the NIA-AA classification. The majority of AD patients showed no TDP-43 pathology beyond the amygdala and therefore had no evidence of significant TDP-43 comorbidity.

Tau and TDP-43 pathology was detected using well-characterized antibodies (i.e. anti-phosphorylated tau, mouse monoclonal PHF1, gift of Professor Peter Davies, Albert Einstein College of Medicine, New York, NY, USA and anti-phosphorylated 409/410 TDP-43, rat monoclonal TAR5P-1D3, gift of Manuela Neumann and Elisabeth Kremmer) and established immunohistochemical methods and neuropathological criteria for scoring as described previously [10]. Postmortem tau and TDP-43 burden were rated on a standard ordinal score (0 = none, 0.5 = rare, 1 = mild, 2 = moderate, 3 = severe) in 16 brain regions (amygdala, hippocampus (CA1), entorhinal cortex, mid-frontal gyrus, angular gyrus, superior-middle temporal gyrus, cingulate gyrus, caudate/putamen, globus pallidus, thalamus, midbrain, substantia nigra, locus coeruleus, pons, cerebellum, medulla) as previously described according to established neuropathological criteria [13]. Cumulative burden across regions (Global tau and Global TDP) was obtained as the sum of these values.

Targeted liquid chromatography mass spectrometry.

We monitored a set of 22 proteotypic peptides corresponding to 9 proteins (Calsyntenin-1, GluA2, GluA4, Neurexin-2a, Neurexin-3a, Neuroligin-2, Syntaxin-1B, Thy-1 and VAMP-2) using the previously described Selected Reaction Monitoring (SRM) method [9]. Briefly, we precipitated the individual CSF samples (100 μl) with cold acetone (-20 ºC overnight) and resuspended in 6 M Urea/ 200 mM ammonium bicarbonate. We reduced the samples with 10 mM dithiothreitol followed by in-solution digestion with LysC (1:10 ratio enzyme:protein, w:w) overnight at 37 °C (650 rpm), followed by an 8 h incubation with trypsin (1:10 ratio enzyme:protein, w:w). The digestion reaction was stopped by adding formic acid (10% total volume). We digested 10 µg of E. Coli protein extract in triplicate in parallel with the samples to evaluate digestion efficiency; 78.2% of the peptide groups showed complete cleavage, 18.7% showed 1 missed cleavage and 3.1% showed more than 1% missed cleavage. Missed cleavages were consistent across triplicates. CSF samples were purified using off-line reverse phase purification. We synthesized custom peptides labeled with ^13^C_6_^15^N_4_ (Arg) or ^13^C_6_^15^N_2_ (Lys) isotopes (Pepotech SRM custom peptides, grade 2 > 70% purity, Thermo Fisher Scientific) corresponding to the synaptic proteins and selected from our previously reported shotgun mass spectromic analysis of CSF [9]). We spiked the isotopically-labeled peptides into each sample as internal standards. The peptide sequence of the internal standards is reported in [13]. Samples (5 µl) were analyzed in a randomized order over a 120-min gradient (0–35% ACN + 0.1% FA) in SRM mode using a triple quadrupole-Qtrap mass spectrometer (5500 QTrap, Sciex, Masachussetts) coupled to a nano-LC chromatography column (300 µl/min, 25-cm C18 column, 75 µm I.d., 2 µm particle size). We visualized and analyzed transitions using Skyline 3.5 as previously described [9]. To evaluate the precision of the internal standards and the stability of the peptides over the course of the experiment, we injected a pool of all the samples over the duration of the mass spectrometric measurements and monitored the peak area of the labeled and endogenous peptides. The % coefficient of variation in light/heavy ratio for each protein was as follows: calsyntenin-1; 4%, gluA2; 60%, gluA4; 9%, neuroligin-2; 12%, neurexin-2a; 7%, neurexin-3a; 6%, syntaxin-1B; 25%, thy-1; 10%, VAMP-2; 26%. Except for GluA2, all proteins gave %CV < 25%. GluA2 was removed from further analyses.

Peak areas for the endogenous (light) and isotopically-labeled (heavy) transitions were extracted and used as input for processing. Using the dataProcess function of MSstats v3.5 package in R [14], light and heavy transitions were log-2 transformed and normalized using the EqualizeMedians function. The light/heavy intensity was calculated for all transitions and summarized using the Tukey’s median polish function. We removed transitions with between-run interference (between RunInterferenceScore < 0.8). All remaining transitions passed the minimum log2 intensity cut-off designated by the MSstats package (7.724). We did not impute missing transition data. One of the three calsyntenin-1 peptides was not detected in any samples and was excluded from further analyses. The final number of peptides corresponding to each protein was as follows: calsyntenin-1 (2), gluA4 (3), neuroligin-2 (2), neurexin-2A (3), neurexin-2A (3), syntaxin-1B (1), thy-1 (3), vamp-2 (1).

### Statistical analysis

Statistical analyses were performed in R version 4.1.2 [15]. Outliers were excluded using the 3 × IQR rule. Where residuals deviated from a Gaussian distribution (Shapiro–Wilk *p* < 0.05), we used square root transformed values, which did not deviate from a Gaussian distribution. Group differences were compared using χ2 test for categorical variables or one-way analysis of covariance (ANCOVA) for continuous variables including sex and age-at-CSF as covariates with post hoc Tukey method. A Kruskal–Wallis test was used to compare Global Tau and TDP-43 burden across male and female participants. We used Spearman rank correlations to determine the relationship between antemortem synaptic protein levels in the CSF and demographic variables. We performed ordinal regression to test for association of synaptic proteins with Tau and TDP-43 scores in individual brain regions, including age-at-death and time from CSF to autopsy as covariates. We performed a mixed entry backward stepwise linear regression (stepAIC function) to identify the best possible predictors of postmortem Global tau and Global TDP-43 (sum of scores across all regions). Age-at-death and time from CSF to death were forced into the model and test variables included all 8 synaptic proteins, biological sex, and AD comorbidity. Akaike Information criteria and variant inflation factors were used to set a limit on the total number of variables included in the final model. The same approach was taken for regressions using MMSE as the outcome measure, with the modification that age-at-CSF and years of education were forced into the model. All reported *r*^*2*^ values were adjusted for the number of predictors in the model. All ANCOVA, Spearman and regression p-values were adjusted for multiple testing of 8 synaptic proteins using the Benjamini–Hochberg method. We assessed the diagnostic utility (area-under-the-curve; AUC) of CSF levels of the synaptic proteins and multimarker panels using receiver operating characteristic (ROC) curves in the pROC package implemented in R. We used the ‘DeLong’ method to compare ROC curves [16].

## Results

### Demographics and clinical data

Table 1 shows the demographic, clinical and neuropathological data for the samples included in the study grouped according to their final neuropathological diagnosis. Forty-five percent of the study participants were female. The FTLD-Tau group had a lower proportion of females (25%) compared to controls (57%, *p* = 0.03), whereas the proportion was comparable across all other groups (*p* > 0.05). There was no difference in years of education across groups (*p* = 0.40). The mean age-at-disease onset (6 year difference, post-hoc *p* = 0.02) and mean age-at-CSF analysis (7 year difference, post-hoc *p* = 0.04) were higher in the AD group compared to FTLD-TDP. The mean age-at-death was higher in AD compared to FTLD-Tau (8 year difference, post-hoc *p* = 0.03) and FTLD-TDP (9 year difference, post-hoc *p* = 0.006). There was no difference in mean disease duration (*p* = 0.07) or time interval between disease-onset and CSF analysis (*p* = 0.79), whereas the mean time interval between CSF analysis and death was longer in the AD group compared to FTLD-Tau (1.4 year difference, post-hoc *p* = 0.04) and FTLD-TDP (2.4 year difference, post-hoc *p* = 0.01). Based on these differences, we included sex and age-at-CSF collection as a covariate in statistical analysis for group comparisons. Eighty-three percent of the FTLD-Tau and 76% of FTLD-TDP showed little to no AD pathology and 72% of the AD patients showed no TDP-43 pathology beyond the amygdala, a proportion that was comparable across groups (*p* = 0.63). The global pathological burden of tau (*p* = 0.66) and TDP-43 (*p* = 0.31) pathology was comparable between male and female participants. Global pathological burden was not associated with the time interval from CSF collection to death (tau; *p* = 0.12, TDP-43; *p* = 0.21) or disease duration (tau; *p* = 0.08, TDP-43; *p* = 0.19) but was inversely related to age-at-death (tau; *p* = 0.04, TDP-43; *p* = 0.02). The mean MMSE score and time interval between MMSE and CSF collection were comparable between FTLD-Tau and FTLD-TDP (both *p* > 0.29).

### Correlation between antemortem CSF levels of the synaptic proteins

**Fig. 1:**
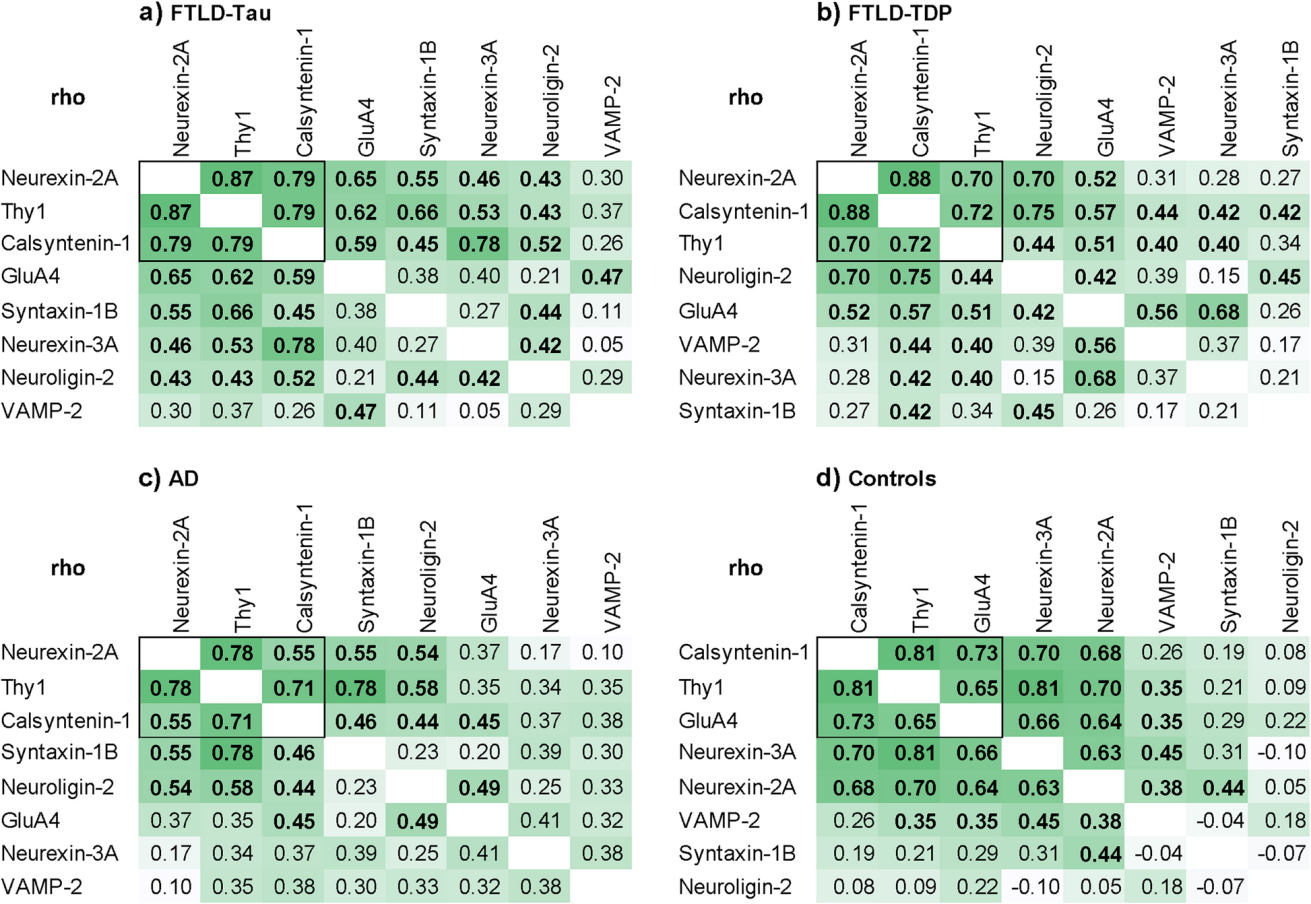
Pair-wise correlation of antemortem CSF levels of 8 synaptic proteins in FTLD, AD and controls. Pair-wise r_s_ coefficients resulting from statistical tests performed in (**a**) FTLD-Tau, (**b**) FTLD-TDP, (**c**) AD and (**d**) controls are shown. Degree of shading is relative to size of Spearman r_s_ coefficients, which are shown in bold where *p* < .05. A set of 3 proteins that correlated in all groups are highlighted by a black box

We first sought to compare the pair-wise correlation between antemortem CSF levels of the synaptic proteins in each neuropathologic group (Fig. 1**)**. A set of 3 proteins (calsyntenin-1, neurexin-2a and thy-1) showed pair-wise correlation in all groups (FTLD-Tau; *r*_*s*_ = 0.79-0.87, *p* < 0.0001, FTLD-TDP; *r*_*s*_ = 0.70-0.88, *p* < 0.009, AD; *r*_*s*_ = 0.55-0.78, *p* < 0.007, Controls; *r*_*s*_ = 0.68-0.81, *p* < 0.0001), while all other protein combinations were group-dependent.

### Antemortem CSF profile of the synaptic proteins across neuropathologic groups

**Fig. 2:**
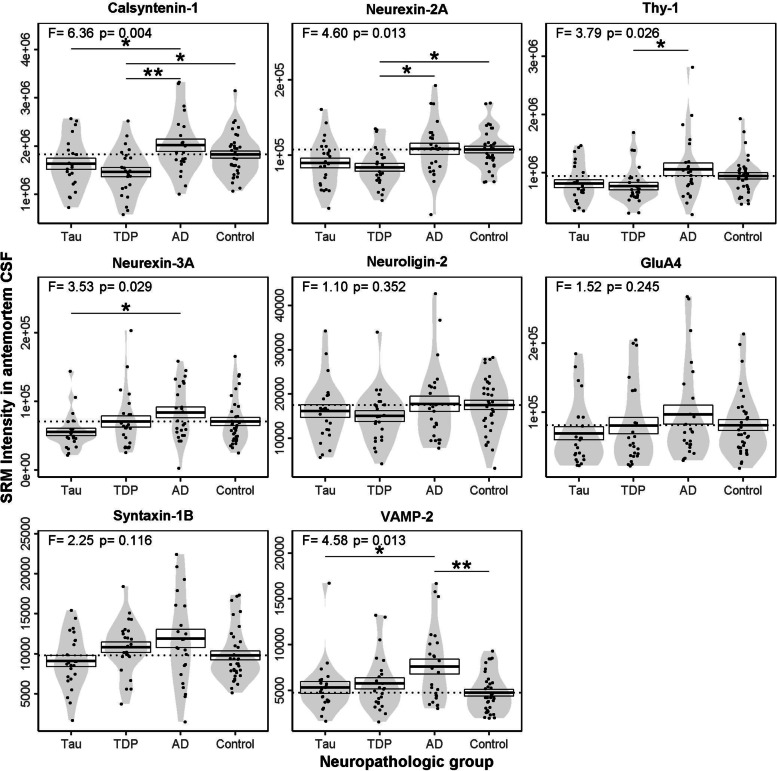
Antemortem CSF SRM intensities of the synaptic panel across patient groups. Violin plots show the distribution of SRM intensities for each synaptic protein quantified in CSF for patients with neuropathological confirmation of FTLD-Tau (Tau), FTLD-TDP (TDP) or AD and cognitively normal subjects (Control). Solid horizontal lines show the mean value for each and boxes represent the standard error of the mean. The horizontal dotted line represents the mean value in the control group for each protein. Summary statistics for ANCOVA including sex and age-at-CSF performed on square root transformed data are shown at the top of each plot. ANCOVA *p*-values are adjusted for multiple testing (9 proteins). *p < .05, **p < .01 for Dunnet’s post-hoc tests

We next compared the CSF profile of the synaptic proteins across neuropathologic groups, including sex and age-at-CSF as covariates (Fig. 2). Of the 8 synaptic proteins, calsyntenin-1 showed the strongest association with neuropathologic group (*F* = 6.36, *p* = 0.004). In FTLD-TDP, we observed low CSF calsyntenin-1 compared to AD (0.72-fold, *p* = 0.001) and controls (0.80-fold, *p* = 0.02). In FTLD-Tau, we observed low CSF calsyntenin-1 compared to AD (0.79-fold, *p* = 0.03) but not controls (0.88-fold, *p* = 0.24). Calsyntenin-1 was comparable between FTLD neuropathological subtypes (*p* = 0.79) and between AD and controls (*p* = 0.60).

Neurexin-2a (*F* = 4.60, *p* = 0.01), thy-1 (*F* = 3.79, *p* = 0.03), neurexin-3a (*F* = 3.53, *p* = 0.03) and VAMP-2 (*F* = 4.58, *p* = 0.01) were also associated with neuropathologic group. Neurexin-2a was lower in FTLD-TDP compared to AD (0.77-fold, *p* = 0.04) and controls (0.78-fold, *p* = 0.02). Thy-1 was lower in FTLD-TDP compared to AD (0.73-fold, *p* = 0.04). Neurexin-3a was lower in FTLD-Tau compared to AD (0.66-fold, *p* = 0.01). VAMP-2 was elevated in AD compared to controls (1.6-fold, *p* = 0.005) and FTLD-Tau (1.43-fold, *p* = 0.03). The association of CSF calsyntenin-1, neurexin-2a and VAMP-2 with patient group remained when sex and age-at-CSF were excluded from the model (*p* = 0.009, *p* = 0.02, *p* = 0.02, respectively).

To determine the influence of AD comorbidity, we repeated these analyses in the FTLD subgroups without significant AD pathology (FTLD-Tau; *n* = 20, FTLD-TDP; *n* = 19). The principal associations we observed across groups in the total dataset held in this subset (Additional file 1). We observed no difference in any of the synaptic proteins between male and female participants (all *p* > 0.21) and no association with age-at-CSF analysis (all *p* > 0.10) in any neuropathologic group. The synaptic proteins were not associated with age-at-disease onset (all *p* > 0.07), duration from disease onset to CSF analysis (all *p* > 0.15), disease duration (all *p* > 0.15) or duration from CSF analysis to autopsy (all *p* > 0.06) in any neuropathologic group.

### Association of antemortem CSF levels of the synaptic proteins with post-mortem pathological burden in FTLD

**Fig. 3:**
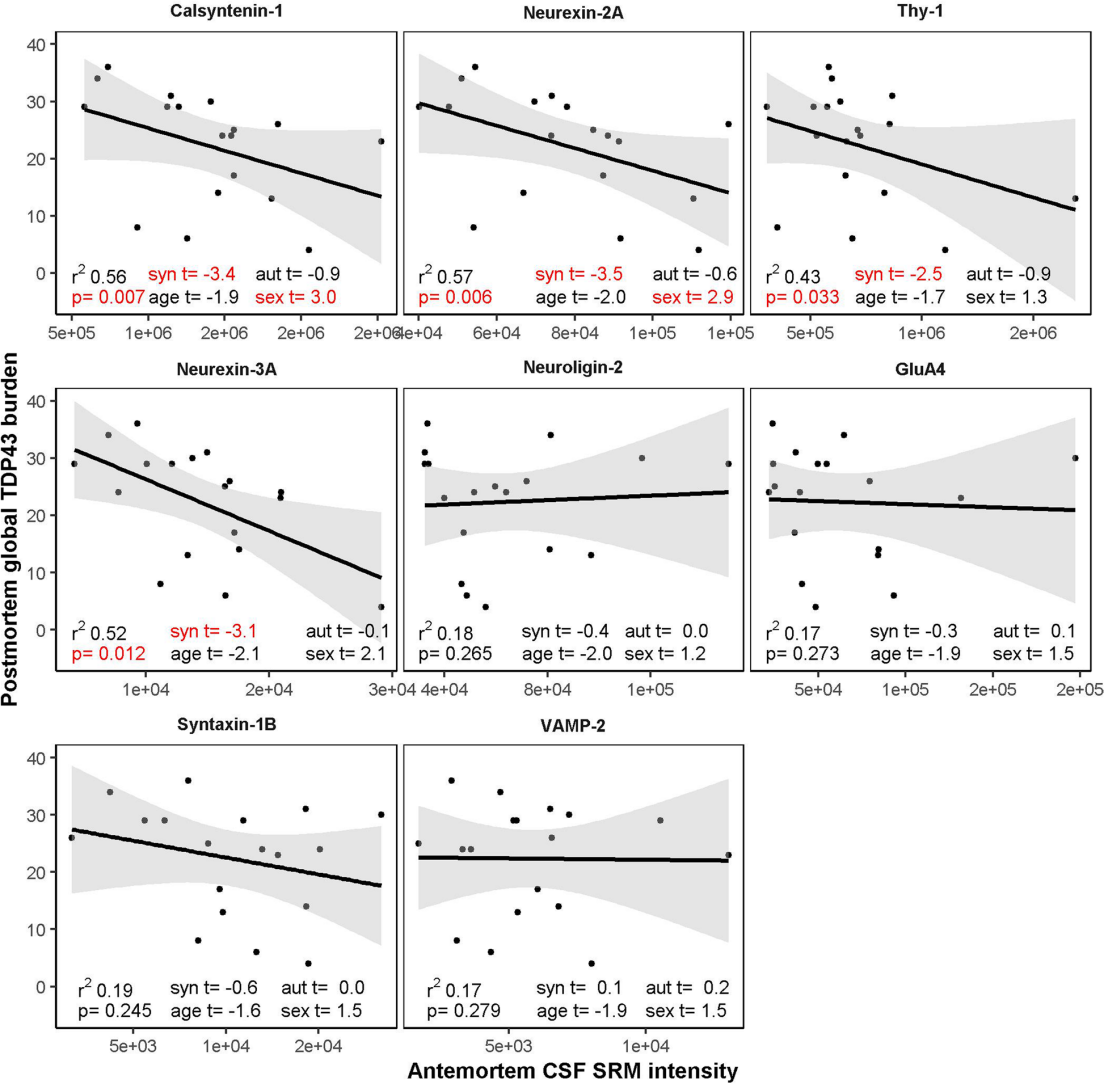
Association of antemortem CSF levels of synaptic proteins with postmortem TDP-43 burden. Scatter plots show the antemortem SRM intensities for selected synaptic proteins quantified in CSF and post-mortem TDP-43 burden for patients with neuropathological confirmation of FTLD. Linear regression lines and standard error (shaded region) are shown for each plot. Adj.r^2^, *p*-values and t-statistics for the synaptic protein (syn t), time from CSF to autopsy (aut t), age-at-death (age t) and biological sex (sex t) are shown for each linear regression. *P*-values were adjusted for multiple testing (9 proteins)

In FTLD-Tau, we observed no association between antemortem CSF levels of the synaptic proteins with age-at-death (all *p* > 0.11). In FTLD-TDP, neurexin-3a was inversely associated with age-at-death (*r*_*s*_ = -0.55, *p* = 0.04). We observed no association between antemortem CSF levels of the 8 synaptic proteins with postmortem global tau burden (Additional File 2; *all p* > *0.17*). Calsyntenin-1, neurexin-2a, thy-1 and neuroligin-2 were inversely associated with postmortem global TDP-43 burden (Fig. 3). With the exception of neuroligin-2 and thy-1, biological sex also significantly contributed to the associations (all *p* = 0.03). Moreover, the inclusion of covariates explained global postmortem TDP-43 burden better than simple linear regression models for each synaptic protein alone (all ANOVA *p* < 0.003). In a secondary analysis, we observed a stronger association of calsyntenin-1 when the outcome measure was restricted to regions with low to medium TDP-43 burden (brainstem + cerebellum + thalamus + basal ganglia; *r*^*2*^ = 0.61; *p* = 0.03). We also observed an association for neurexin-3a in regions with low to medium TDP-43 burden (*r*^*2*^ = 0.69; *p* = 0.01). This may be due to a ceiling effect in regions with high burden.

To determine whether a combination of synaptic proteins could better explain postmortem tau and TDP-43 burden, we performed stepwise mixed entry linear regression starting with the synaptic proteins and covariates. No multimarker model was associated with global Tau (*p* > 0.13). On the other hand, a 6 variable model including calsyntenin-1, neurexin-3a, VAMP-2 and all 3 covariates was associated with global TDP-43 burden (Table 2a; *r*^*2*^ = 0.69, *p* = 0.003). Age-at-death, biological sex, calsyntenin-1, VAMP-2 were the significant contributors to this model (henceforth named Panel A[CLSTN1-VAMP2]). When controlling for the other variables, lower age-at-death (*p* = 0.01), lower CSF calsyntenin-1 (*p* = 0.0006), elevated CSF VAMP-2(*p* = 0.02) and being female (*p* = 0.01) were associated with elevated global TDP-43 protein. Additional File 3 plots the relative regression line of each variable.

**Table 1:** Demographic, clinical and neuropathological data for the samples included in the study

N	FTLD-Tau	FTLD-TDP	AD	Control	*p*-value
	24	25	25	35	
**Female, n (%)**	6 (25)	13 (52)	10 (40)	20 (57)	.08
**Education (years)**	15.6 (3.5)	15.1 (2.7)	14.8 (3.1)	15.7 (3.4)	.40
**Age-at-disease onset (years)**	64 (9.3)	63 (9.0)	69 (5.6)	NA	.02^a^
**Age-at-CSF analysis (years)**	67 (11.3)	66 (8.7)	73 (5.7)	69 (7.3)	.04^b^
**Age-at-death (years)**	71 (12.6)	70 (9.5)	79 (6.0)	NA	.005^c^
**Time interval, onset–CSF (years)**	3.7 (2.7)	3.7 (2.7)	3.6 (1.6)	NA	.79
**Time interval, CSF–death (years)**	4.6 (3.6)	3.6 (3.0)	6.0 (2.3)	NA	.01^d^
**Total disease duration, onset–death (years)**	8.4 (4.5)	7.2 (3.4)	9.6 (3.0)	NA	.07
**Participants without comorbidity, n (%)**	20 (83)	19 (76)	18 (72)	NA	.63
**Global Tau pathology**	29.3 (12.0)	10.0 (8.0)	NA	NA	< .0001
**Global TDP pathology**	9.7 (10.1)	26.1 (6.8)	NA	NA	.0002
**MMSE score**	24.0 (5.2)	20.9 (6.8)	NA	NA	.29
**Time interval, CSF–MMSE (months)**	0.7 (1.2)	0.7 (0.8)	NA	NA	.84

Unless otherwise specified, the results are expressed as mean (SD). Global tau and TDP pathology refers to sum of ordinal values across 16 brain regions. Comorbidity refers to FTLD without incidental AD pathology (NIA-AA stage ≤ B1) and AD without TDP-43 pathology beyond the amygdala. *P*-values were obtained from χ2 test, one-way ANOVA with post-hoc Tukey or Kruskal–Wallis rank-sum tests with post-hoc Dunn’s test. Post-hoc tests where adj*.p* < *.*05 are as follows

^a^FTLD-TDP vs AD; *p* = .02

^b^FTLD-TDP vs AD; *p* = .04

^c^FTLD-TDP vs AD; *p* = .03, FTLD-TDP vs AD; *p* = .006

^d^FTLD-TDP vs AD; *p* = .04, FTLD-TDP vs AD; *p* = .01

*MMSE* Mini-mental state examination

**Table 2:** Association of step-wise linear regression models with postmortem TDP-43 burden and MMSE outcome measures and diagnostic accuracy

		**Summary Statistics**					**Forced Entry**		**Test variables**													
**Panel**	**Study group**	**AIC**		**adj.r**^**2**^	**p**		**CSF-autopsy (a) / Education (b)**	**Age-at-death (a) / Age-at-CSF (b)**	**AD comorbidity**		**Sex**	**Calsyntenin-1**	**VAMP-2**	**Neurexin-3a**		**GluA4**	**Syntaxin-1b**		**Neuroligin-2**		**Neurexin-2a**	**Thy-1**
**a) Outcome measure: Global TDP-43**																						
A[CLSTN1-VAMP2]	FTLD	119		0.69	**0.003**		-0.91	**-2.91**			**3.1**	**-4.8**	**2.71**	-1.28								
**b) Outcome measure: MMSE**																						
A[CLSTN1-VAMP2]	FTLD	273		0.19	**0.03**		1.21	-1.9				-0.36	**2.41**	**-2.43**								
B[CLSTN1-VAMP2-GLUA4]	FTLD-Tau	98		0.49	**0.04**		-1.79	-2.45				**-3.60**	**3.15**			**2.57**	1.90					
	FTLD-TDP	174		0.02	0.40		0.93	-1.46				-0.23	0.80			0.91	-0.68					
	FTLD	279		0.09	0.16		1.46	-1.29				-0.65	1.16			0.99	-1.29					
C[GLUA4-NRX3A-STX1B]	FTLD-Tau	131		0.02	0.45		1.49	**-0.55**	**0.22**					**-1.59**		**1.28**	-0.57		**0.65**			
	FTLD-TDP	162		0.40	**0.02**		**2.54**	**-3.21**	1.33					**-3.42**		**3.71**	**-2.4**		1.69			
	FTLD	291		0.30	**0.004**		**2.11**	**-2.53**	1.12					**-3.5**		**3.74**	**-2.22**		1.22			
**c) Outcome measure: AUC**																						
	**FTLD-Tau vs Controls**				**FTLD-Tau vs AD**				**FTLD-TDP vs Controls**			**FTLD-TDP vs AD**						**FTLD-TDP vs FTLD-Tau**				
**Model**	**AUC**		**95%CI**		**AUC**	**95%CI**			**AUC**	**95%CI**		**AUC**			**95%CI**			**AUC**		**95%CI**		
A[CLSTN1-VAMP2]	71.1		56–85		80.1	66–92			76.9	64–88		77.2			63–89			72.6		57–86		
B[CLSTN1-VAMP2-GLUA4]	71.3		56–85		75.8	60–89			82.3	71–93		81.3			68–92			83.0		70–94		
C[GLUA4-NRX3A-STX1B]	64.6		49–79		79.7	66–91			65.8	52–79		66.7			51–82			70.8		55–84		

Summary statistics and composition of each step-wise regression model is shown for models associated with postmortem TDP-43 burden and MMSE outcome measures (a-b) and their diagnostic performance in FTLD and FTLD neuropathologic subtypes (c)

The model statistics and effect size for each variable included in the final regression model resulting from backward step-wise regression as predictors for the outcome variable are given for models where *p* < 0.05

Variables that significantly contributed to the model (*p* < 0.05) are shown in bold

*AIC* Akaike Information criteria

### Association of antemortem CSF levels of the synaptic proteins with cognitive performance in FTLD

We first tested the association of the synaptic proteins from Panel A[CLSTN1-VAMP2] with MMSE score in FTLD patients including sex, age-at-CSF, and years of education as covariates. The panel was associated with MMSE in the total FTLD group (Table 2b; *r*^*2*^ = 0.19, *p* = 0.03) but not in FTLD-Tau (*r*^*2*^ = 0.33, *p* = 0.09) or FTLD-TDP (*r*^*2*^ = 0.17, *p* = 0.13). For comparison, no individual synaptic protein was associated with MMSE in FTLD (all *p* > 0.48) or in either neuropathologic subtype (FTLD-Tau; *p* = 0.35, FTLD-TDP; *p* > 0.52). Neurexin-3a and VAMP-2 were the significant contributors to this model. When controlling for the other variables, lower CSF VAMP-2 (*p* = 0.02) and elevated CSF neurexin-3a (*p* = 0.02) were associated with worse MMSE score. Additional file 4 plots the relative regression line of each variable.

To determine whether other combinations of synaptic proteins could improve the association of Panel A, we performed stepwise linear regression starting with the synaptic proteins and covariates. Compared to Panel A, a 6 variable model including calsyntenin-1, gluA4, syntaxin-1b, VAMP-2, age-at-CSF and education showed a stronger association with MMSE in FTLD-Tau (Table 2b; *r*^*2*^ = 0.49, *p* = 0.04). Age-at-CSF, calsyntenin-1, VAMP-2 and gluA4 were the significant contributors to the model (henceforth termed Panel B[CLSTN1-VAMP2-GLUA4]). When controlling for the other variables, elevated age (*p* = 0.03), elevated CSF calsyntenin-1 (*p* = 0.005), lower CSF VAMP-2 (*p* = 0.01) and lower CSF gluA4 (*p* = 0.03) were associated with worse MMSE score. Additional file 5 plots the relative regression line for each variable. An alternative 6 variable model, including gluA4, neuroligin-2, neurexin-3a, syntaxin-1b, age-at-CSF and education, showed a stronger association with MMSE in FTLD-TDP (Table 2b; *r*^*2*^ = 0.38, *p* = 0.02) and in all FTLD (Table 2b; *r*^*2*^ = 0.30, *p* = 0.002). GluA4, neurexin-3a and syntaxin-1b, age-at-CSF and years of education were the significant contributors to this model (henceforth termed Panel C[GLUA4-NRX3A-STX1B]). When controlling for the other variables, less education (*p* = 0.02), elevated age (*p* = 0.005), lower CSF gluA4 (*p* = 0.002), elevated neurexin-3a (*p* = 0.003) and elevated CSF syntaxin-1b (*p* = 0.03) were associated with worse MMSE score. Additional file 6 plots the relative regression line for each variable.

Thus, several multimarker panels of synaptic proteins are associated with cognitive performance in FTLD. Panels B and C were not associated with postmortem global TDP-43 or global Tau burden (all *p* > 0.1).

Diagnostic value.

The synaptic proteins showed modest diagnostic accuracy to distinguish FTLD-Tau from controls (highest AUC = 0.67 for neurexin-2a). The best multimarker panel was Panel B[CLSTN1-VAMP2-GLUA4], which showed only a nominal improvement (Table 2c; AUC = 0.71) compared to neurexin-2a (*p* = 0.67). This panel was statistically comparable to tau CSF biomarkers, pTau, tTau and pTau/tTau ratio (AUC = 0.65–73, all *p* > 0.53 vs Panel B). When distinguishing FTLD-Tau from AD, neurexin-3a gave nominally the highest AUC (0.75, 95% CI 0.60-0.88). Panel A[CLSTN1-VAMP2] showed a nominal improvement (Table 2c; AUC = 0.80) compared to neurexin-3a (*p* = 0.61) but still performed worse than pTau and the ptau/Aβ42 ratio (AUC = 0.95–98, all *p* < 0.04 vs Panel A).

Neurexin-2a gave nominally the highest AUC (0.75, 95% CI 0.62-0.87) for distinguishing FTLD-TDP from controls. Panel B[CLSTN1-VAMP2-GLUA4] showed only a nominal improvement (Table 2c; AUC = 0.82) compared to neurexin-2a (*p* = 0.41) but performed better than CSF tau markers (AUC = 0.62–68, all *p* < 0.04 vs Panel B). When distinguishing FTLD-TDP from AD, calsyntenin-1 gave nominally the highest AUC (0.76, 95% CI 0.62-0.89). Panel B showed only a nominal improvement (Table 2c; AUC = 0.81) compared to calsyntenin-1 (*p* = 0.56) and was significantly worse than the pTau/Aβ42 ratio (AUC = 0.96, *p* < 0.03 vs Panel B).

None of the synaptic proteins alone could discriminate FTLD-TDP from FTLD-Tau subtypes (all AUC < 0.67). However, Panel B performed significantly better (Table 2c; AUC = 0.83) than tau markers (0.51–62, all *p* < 0.05 vs Panel B) and the ptau/aβ42 ratio (AUC = 0.56, *p* = 0.009).

## Discussion

Here we report a comprehensive evaluation of the antemortem CSF levels of 8 synaptic proteins in a neuropathological cohort of FTLD (FTLD-Tau and FTLD-TDP), AD and cognitively normal controls. CSF levels of calsyntenin-1 and neurexin-2a were correlated in all patient groups and were lower in the FTLD-TDP neuropathological subtype compared to neuropathologically confirmed AD and cognitively normal controls. Furthermore, antemortem CSF levels of calsyntenin-1 and neurexin-2a inversely correlated with global TDP-43 burden post-mortem. Combining calsyntenin-1 with VAMP-2 and neurexin-3a improved this association with TDP-43 burden and was also correlated with cognitive performance in FTLD. Combining calsyntenin-1 with VAMP-2, gluA4 and syntaxin-1b showed high accuracy in the differential diagnosis of FTLD-TDP and FTLD-Tau neuropathologic subtypes and was associated with cognitive performance in FTLD-Tau. Thus, when measured in CSF, combinations of multiple synaptic proteins were better predictors of global TDP burden and cognitive performance and showed better diagnostic accuracy compared to individual synaptic proteins. To our knowledge, this is the first study to relate CSF levels of synaptic proteins to FTLD neuropathological subtypes.

Calsyntenin-1 is abundant in most neurons of the CNS [36] where it has been shown to modulate postsynaptic calcium signaling and promote dendritic spine assembly [15]. VAMP-2 and syntaxin-1b are synaptic vesicle recycling proteins predominantly found at glutamatergic synapses [17] as part of the synaptic exocytosis core vesicular complex [18] where they regulate the releasable pool of glutamate at the pre-synapse [19]. GluA4 is the predominant AMPA receptor subunit in PV interneurons [44]. The neurexins are a complex family of proteins that are generated from three different genes (*NRXN1*, *NRXN2*, *NRXN3*) with alternative promoters (α, β, γ) and extensive alternative splicing resulting in over 1000 distinct neurexin isoforms, each associated with specific neuronal cell types [20]. The formation of a trans-synaptic complex with their post-synaptic partners, the neuroligins, allows the structural assembly of excitatory synapses, by triggering the recruitment of postsynaptic NMDA and AMPA glutamate receptors [21, 22]. The composition of these panels thus support the growing evidence for involvement of the glutamatergic system in FTLD syndromes [23].

A previous study has reported an association between low CSF calsyntenin-1 levels in patients with behavioral variant FTD (bvFTD) compared to cognitively normal controls and compared to presymptomatic carriers of *C9orf72*, *GRN* or *VCP* mutations [24]. As the pathology underlying clinically defined FTLD syndromes is heterogeneous, the results of the previous study cannot be directly compared to the current study; clinical diagnoses of bvFTD is split between FTLD-TDP and FTLD-Tau subtypes and the small number of cases with a bvFTD diagnosis in the current study (*n* = 10) prohibits meaningful comparison across syndromes. Nevertheless, both studies point towards altered abundance of calsyntenin-1 in CSF from patients clinically diagnosed with FTLD syndromes and those with neuropathologically confirmed FTLD compared to cognitively normal controls. Neurexins have also been shown to facilitate the calcium-dependent release of synaptic vesicles [25, 27, 28].

Another important finding of this study is that while AD neurofibrillary tangle pathology (Braak score < 2 [31]) was present in 17% of the FTLD-Tau and 24% of the FTLD-TDP patients, stepwise regression showed that AD comorbidity was not a good predictor of TDP-43 burden and was therefore excluded from the final model. On the other hand, AD comorbidity was a predictor of MMSE and was included in Panel C. Thus, AD comorbidity may influence cognitive performance in FTLD. This is consistent with a recent study showing that neurodegenerative comorbidities contribute to transition from mild cognitive impairment to dementia [29].

### Study limitations

A limitation of this work is the relatively small study size compared to other CSF biomarker studies. However, CSF samples from well-annotated autopsy cases of these uncommon conditions are scarce and the neuropathological confirmation reduces the substantial heterogeneity inherent in clinical FTD cohorts. It should also be noted that the mean time from CSF to death was 5 years in FTLD-Tau and 4 years in the FTLD-TDP subtypes, reaching up to 10 years in some cases. This long interval and variability could influence the relationship between participants’ CSF biochemical signature and their final neuropathological findings. Finally, our control participants lack neuropathological confirmation. However, complete clinical and neuropsychological evaluations were performed to exclude significant medical (and specifically neurological) conditions in these participants.

## Conclusions

A biomarker that can distinguish FTLD neuropathologic subtypes in clinical cohorts would be a valuable addition to the biomarker arsenal. In this regard, Panel B[CLSTN1-VAMP2-GLUA4] identified in this study provides proof-of-concept that these synaptic proteins are differentially altered and can distinguish between the two most common neuropathologic subtypes. Moreover, all panels described here were associated with MMSE score and therefore show proof-of-concept as surrogate measures of cognitive performance. These panels may be relevant for future clinical trials targeting TDP-43 or tau pathology, where there will be a need for a surrogate measure of cognitive performance, independent of the drug target. In addition, the postsynaptic modulator, calsyntenin-1 both alone and in combination with other synaptic proteins, has potential as an objective measure of TDP-43-mediated degeneration in FTLD. Validation of these findings in independent cohorts is needed to fully determine their clinical potential.

## Supplementary Information

**Additional file 1.**

**Additional file 2.**

**Additional file 3.**

**Additional file 4.**

**Additional file 5.**

**Additional file 6.**

## Data Availability

The data analyzed during the current study are available from the corresponding author on reasonable request.

## Abbreviations

AD: Alzheimer's disease
ANCOVA: Analysis of Covariance
AUC: Area-under-the-curve
bvFTD: Behavioural variant
CSF: Cerebrospinal fluid
FTD: Frontotemporal dementia
FTLD: Frontotemporal lobar degeneration
ROC: Receiver operating characteristic
SRM: Selected Reaction Monitoring
TDP-43: Transactive response DNA-binding protein-43
