# Supplementary figures and images for: Multimarker synaptic protein cerebrospinal fluid panels reflect TDP-43 pathology and cognitive performance in a pathological cohort of frontotemporal lobar degeneration
Source: Mol Neurodegener. 2022 Apr 8;17:29. doi: 10.1186/s13024-022-00534-y (PMC8991834; doi:10.1186/s13024-022-00534-y)

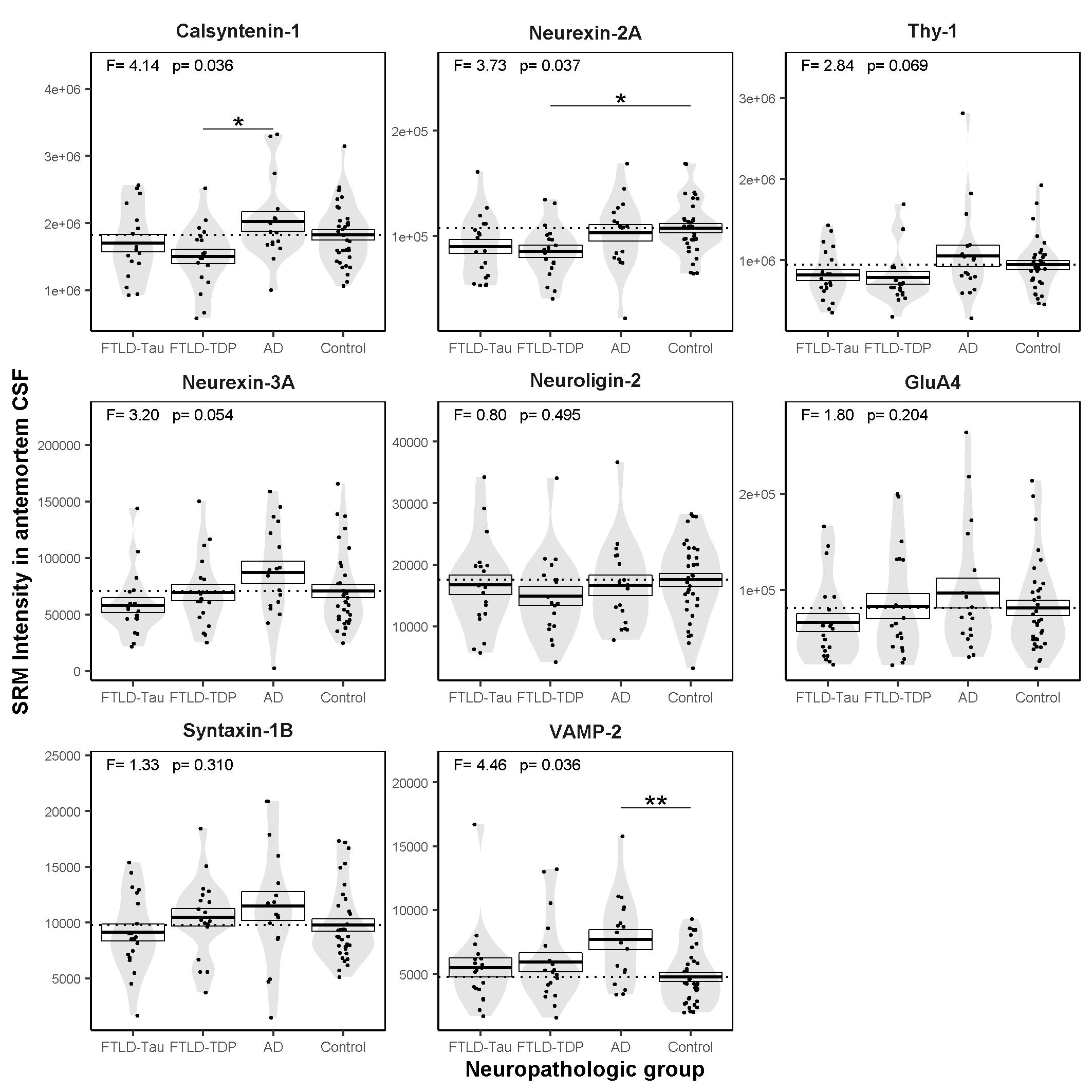

Supplement: Supplementary file 1 — Additional file 1. [file 13024_2022_534_MOESM1_ESM.jpeg]

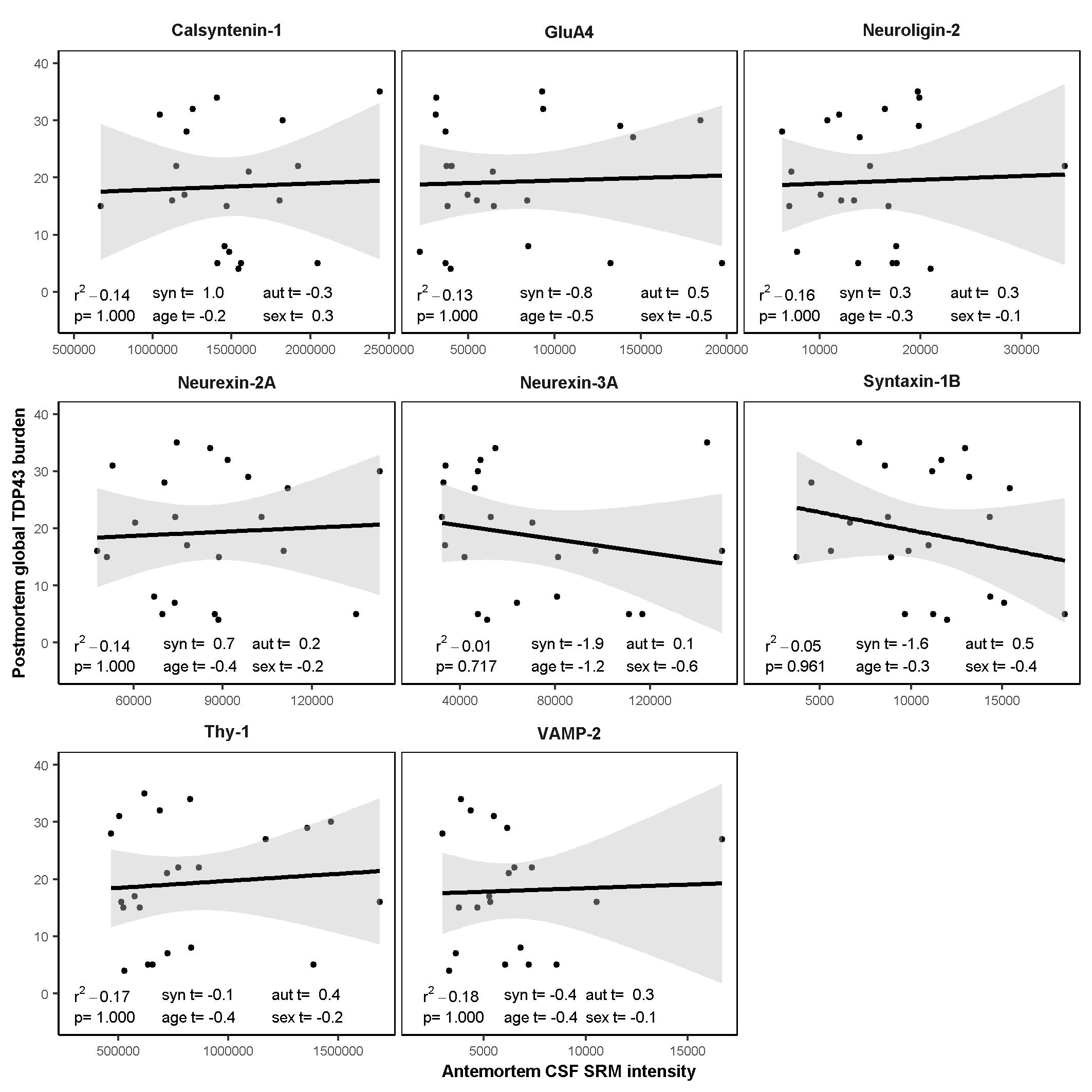

Supplement: Supplementary file 2 — Additional file 2. [file 13024_2022_534_MOESM2_ESM.jpeg]

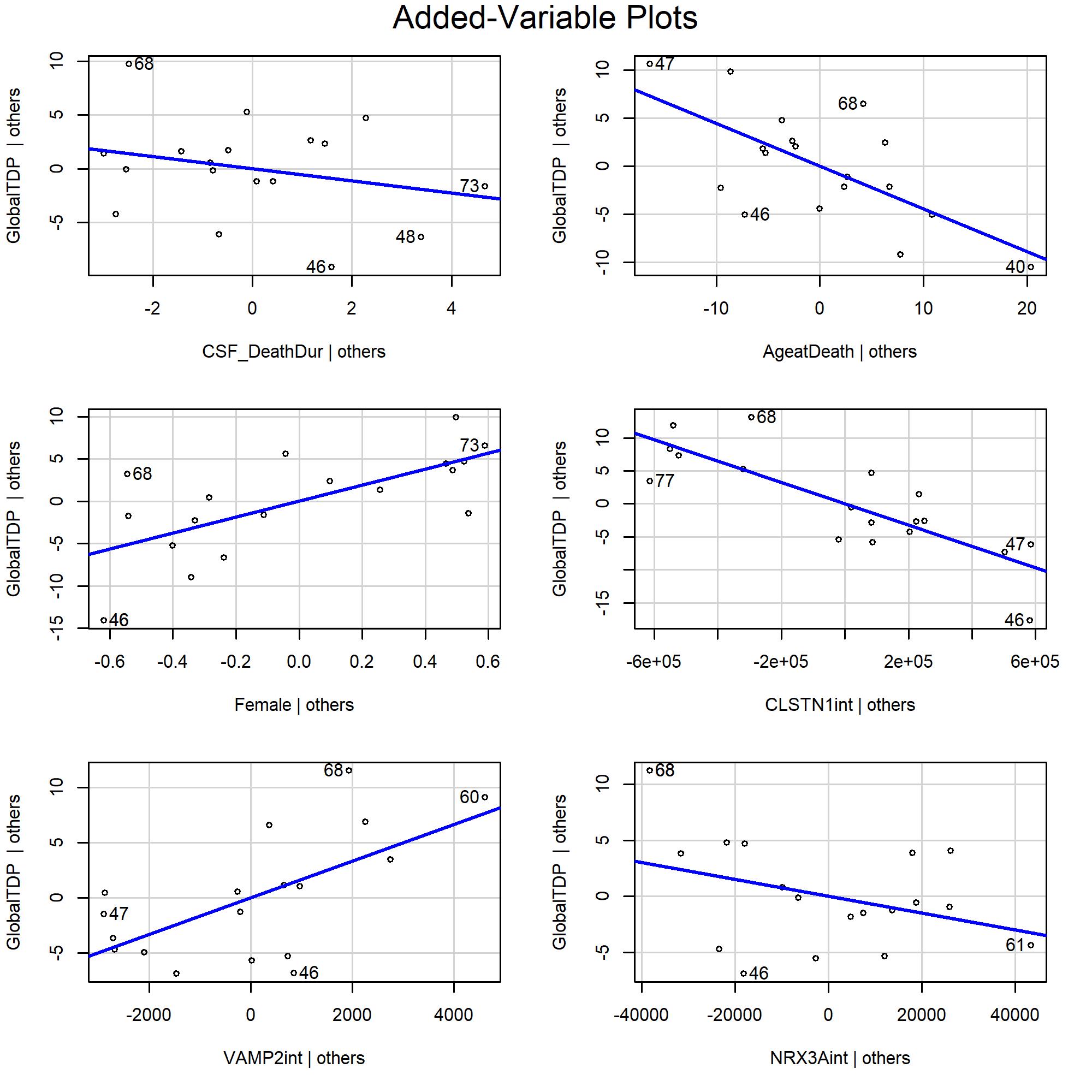

Supplement: Supplementary file 3 — Additional file 3. [file 13024_2022_534_MOESM3_ESM.jpeg]

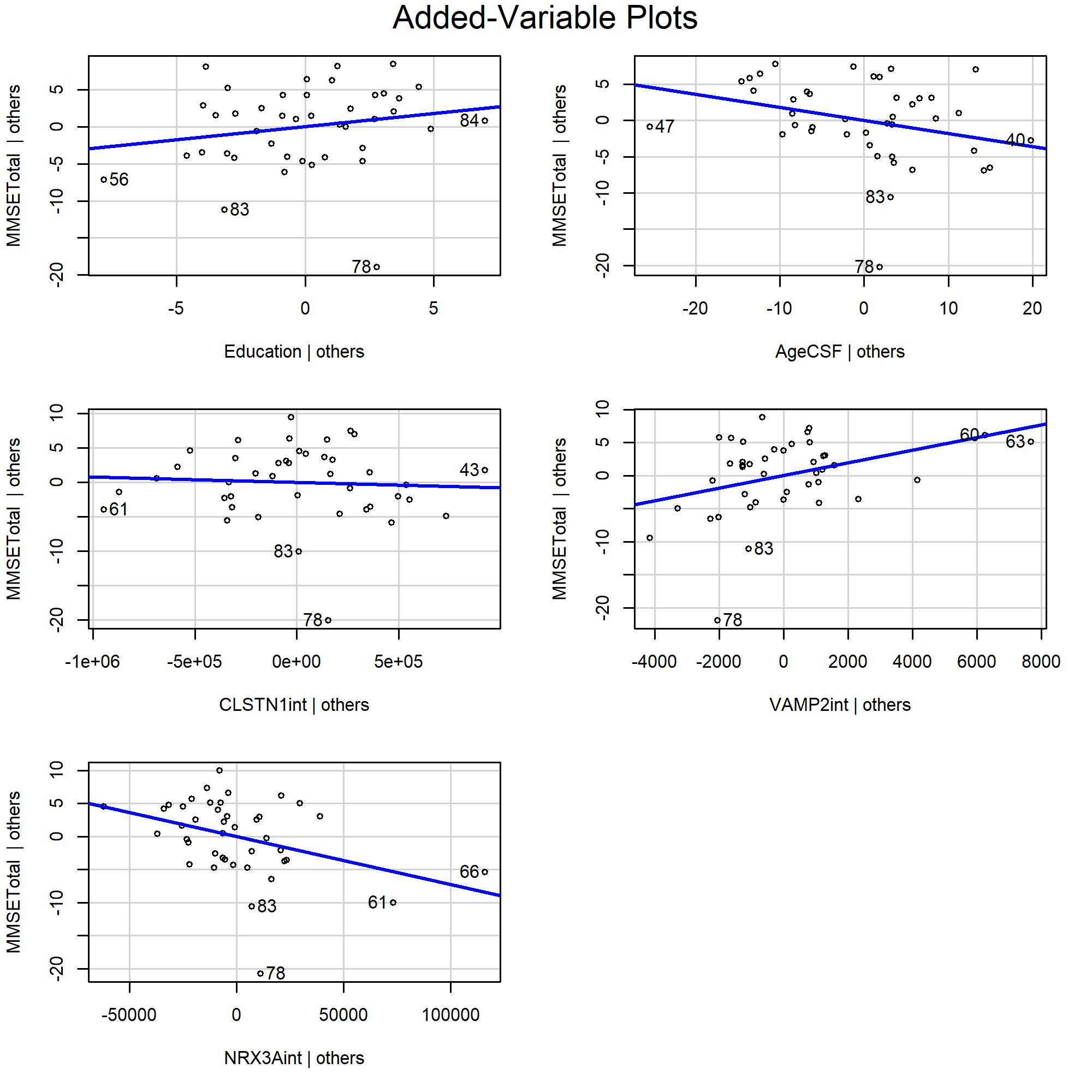

Supplement: Supplementary file 4 — Additional file 4. [file 13024_2022_534_MOESM4_ESM.jpeg]

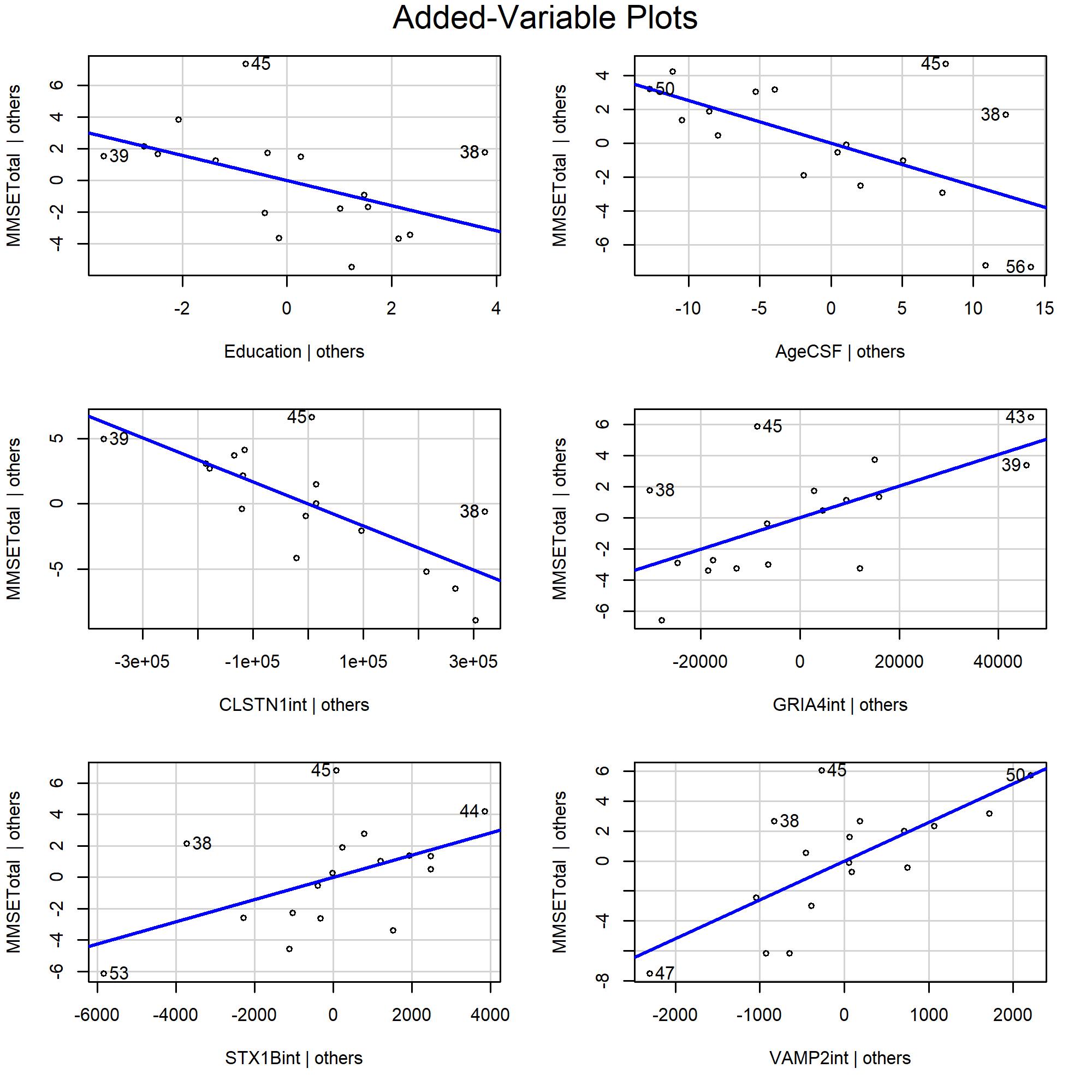

Supplement: Supplementary file 5 — Additional file 5. [file 13024_2022_534_MOESM5_ESM.jpeg]

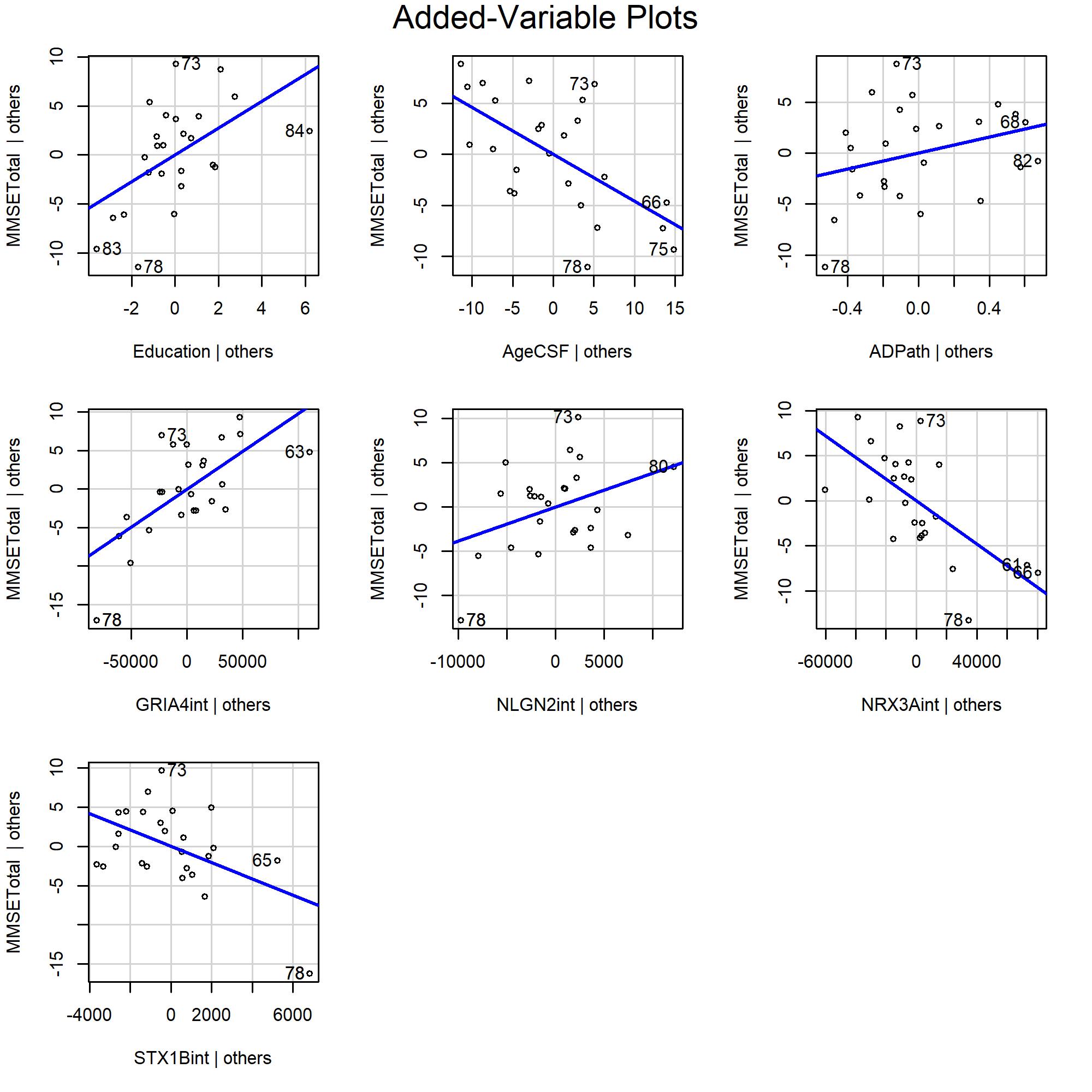

Supplement: Supplementary file 6 — Additional file 6. [file 13024_2022_534_MOESM6_ESM.jpeg]
